# A Blue Native-PAGE analysis of membrane protein complexes in *Clostridium thermocellum*

**DOI:** 10.1186/1471-2180-11-22

**Published:** 2011-01-26

**Authors:** Yanfeng Peng, Yuanming Luo, Tingting Yu, Xinping Xu, Keqiang Fan, Youbao Zhao, Keqian Yang

**Affiliations:** 1 State Key Laboratory of Microbial Resources, Institute of Microbiology, Chinese Academy of Sciences, Beijing 100101, PR China; 2 Department of physiology and Biophysics, School of Medicine, Virginia Commonwealth University, 1101 East Marshall Street, Richmond, VA 23298, USA

## Abstract

**Background:**

*Clostridium thermocellum *is a Gram-positive thermophilic anaerobic bacterium with the unusual capacity to convert cellulosic biomass into ethanol and hydrogen. Identification and characterization of protein complexes in *C. thermocellum *are important toward understanding its metabolism and physiology.

**Results:**

A two dimensional blue native/SDS-PAGE procedure was developed to separate membrane protein complexes of *C. thermocellum*. Proteins spots were identified by MALDI-TOF/TOF Mass spectrometry. 24 proteins were identified representing 13 distinct protein complexes, including several putative intact complexes. Interestingly, subunits of both the F1-F0-ATP synthase and the V1-V0-ATP synthase were detected in the membrane sample, indicating *C. thermocellum *may use alternative mechanisms for ATP generation.

**Conclusion:**

Two dimensional blue native/SDS-PAGE was used to detect membrane protein complexes in *C. thermocellum*. More than a dozen putative protein complexes were identified, revealing the simultaneous expression of two sets of ATP synthase. The protocol developed in this work paves the way for further functional characterization of these protein complexes.

## Background

*Clostridium thermocellum *is a Gram-positive thermophilic anaerobe capable of degrading cellulose and producing ethanol and hydrogen. These qualities render *C. thermocellum *potentially useful for the production of biofuel from biomass. The cellulytic activities of this organism were well studied, the corresponding enzymes were found to organize into a cell surfaced bound multienzyme complex, termed cellulosome [1]. The arrangement of the enzymatic subunits in the cellulosome complex, made possible by a scaffoldin subunit, promotes enhanced substrate binding and degradation. However, other parts of its cellular functions are not well understood. Recently, a genome scale metabolic model was constructed [2], which provides a good basis for the overall understanding of its metabolism. Since membrane is where many important physiological functions, such as energy generation, protein trafficking, and small molecule transport [3], take place, we focused on membrane protein complexes as a start point to identify unique features of *C. thermocellum*. Identification of protein complexes in *C. thermocellum *is an important step toward understanding cellular behavior at an integrative level.

Blue native-PAGE (BN-PAGE) is a charge shift method first developed by Schägger and von Jagow [4] to separate membrane protein complexes. It has been used successfully to characterize respiratory complexes in yeast mitochondria and *Paracoccus denitrificans *[5,6], photosynthetic complexes in plants and *Synechocystis *[7,8], and cell envelope protein complexes in *E. coli *[9,10]. It differs from other native gel electrophoresis mainly because the electrophoretic mobility of a protein is determined by the negative charge of the bound Coomassie blue dye, while separation of proteins is achieved by the molecular sieve effect provided by the polyacrylamide gradient of descending pore size similar to other PAGE methods. BN-PAGE, when coupled with a second dimensional SDS-PAGE and mass spectrometry offers an attractive proteomic solution for analysis of membrane protein complexes and for basic expression profiling. It can complement traditional two-dimensional gel electrophoresis proteomic method by providing a platform to separate membrane proteins.

In this work, we developed a BN-PAGE protocol for the analysis of membrane protein complexes of *C. thermocellum*.

## Results and Discussion

### Preparation of Membrane Protein Samples

Purification of protein complexes in an intact form (*i.e. *complete with all peripherally associated proteins) is largely dependent on the solubilization conditions used and can differ for various complexes. By testing four commonly used detergents at different concentrations (see "Methods"), we were able to select a protocol using the detergent n-dodecyl-D-maltoside (DDM). This protocol detected a number of complexes in the molecular mass range from 60 to over 1,000 kDa. The molecular mass of protein complexes was calculated by plotting the MWs of marker proteins against their migration distances.

**Figure 1:**
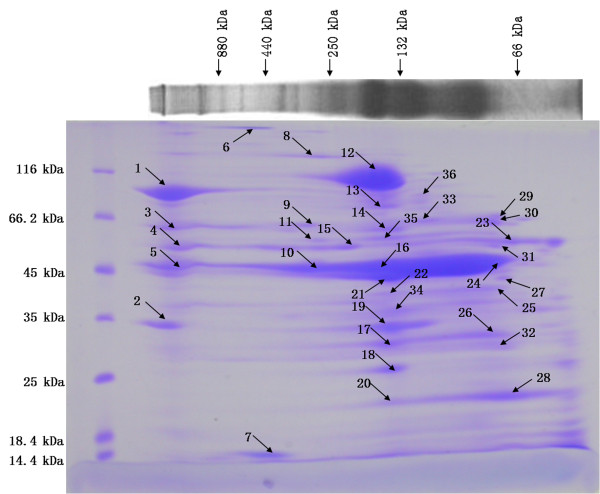
**Coomassie blue-stained 2D BN/SDS-PAGE separation of membrane protein complexes of *C. thermocellum***. Approximately 40 μg of protein was loaded in the first dimensional BN-PAGE lane. Sizes of molecular mass markers are indicated on the top of BN-P|AGE gel and at the left of the SDS gel. The slice of first dimensional BN-PAGE separation gel was placed on top of the second dimensional SDS-PAGE gel and resolved. Protein spots picked for mass spectrometry analysis are marked by arrows and numbered.

To identify the individual proteins in each complex, the one-dimensional BN gel strips were analyzed in the second dimension by SDS-PAGE, Figure 1. Putative complexes were consequently resolved into vertical "channels" enabling visualization of the individual constituents. Proteins that had formed a complex in the BN gel were tentatively recognized by their locations on a vertical line on the SDS gel, and also by their similar shapes on the SDS gel (as a result of co-migration in the BN gel).

### Protein Identification

Thirty six spots were picked from the SDS gel for MALDI-TOF/TOF identification. Thirty proteins were identified in 28 spots (Figure 1), and they represent 24 different proteins (Table 1). Among them, 9 proteins were predicted by TMHMM [11,12] (transmembrane hidden Markov model, http://www.cbs.dtu.dk/services/TMHMM/) to be membrane protein containing α-helical transmembrane segments. The rest maybe membrane-associated proteins (described below). Many atypical membrane proteins are tethered to the membranes through lipid moieties, hydrophobic patches, charge interactions or by their association with a membrane protein complexes. The identified proteins were organized into functional groups based on COG using COGnitor tool available at NCBI [13,14] and transporter related proteins were organized in membrane transporter complexes. Putative protein complexes and their estimated sizes observed on the BN-PAGE were summarized in Table 2. The false positive rate of protein identification was calculated by reverse database search to be lower than 2.5%.

**Table 1:** Putative membrane proteins of *C. thermocellum *identified by mass spectrometry

**Spots Id**^***a***^	Gene locus	NCBI accession number/gi	**Protein descriptions**^***b***^	**Mr**^***c***^	MASCOT Score	Peptides matched	Sequence Coverage (%)	TMHMM prediction
1	Cthe_0423	125972944	bifunctional acetaldehyde/alcohol dehydrogenase	95992.3	427	46	39	
2	Cthe_0858	125713600	hypothetical protein	35296.4	411	26	58	1
3	Cthe_2253	125974738	ATP-dependent metalloprotease FtsH	66652.9	253	34	45	2
4	Cthe_0699	125713442	carboxyl transferase	56037.9	700	39	49	
5	Cthe_1020	125973535	solute-binding protein	49976.2	164	28	45	
6	Cthe_0016	125972541	Ferritin and Dps	18602.9	61	9	42	
7	Cthe_0016	125972541	Ferritin and Dps	18602.9	189	14	42	
8	Cthe_2693	125975175	hypothetical protein	17817.5	74	12	26	1
9	Cthe_2267	125714977	V-type ATP synthase subunit A	65320	214	32	33	
10	Cthe_1020	125973535	solute-binding protein	49976.2	199	25	44	
10	Cthe_2268	125714978	V-type ATP synthase beta chain	50714.2	109	26	43	
10	Cthe_2608	125975091	ATP synthase F1, beta subunit	51000	87	22	38	
11	Cthe_2606	125975089	ATP synthase F1, alpha subunit	55810	307	22	33	
12	Cthe_2348	125715058	S-layer-like region; Ig-related	113309.3	550	42	34	1
13	Cthe_0418	125972939	polynucleotide phosphorylase/polyadenylase	77304	84	17	26	
14	Cthe_3148	125975626	ABC transporter related protein	70461.1	95	12	16	5
15	Cthe_0699	125973217	carboxyl transferase	56037.9	148	25	38	
16	Cthe_1020	125973535	solute-binding protein	49976.2	486	33	48	
17	Cthe_1557	125974066	ABC transporter related protein ATP-binding protein	30203.7	175	21	47	
18	Cthe_1018	125973533	binding-protein-dependent transport systems inner membrane component	31919.9	67	13	23	6
19	Cthe_1840	125974344	cysteine synthase	33392	469	25	57	
20	Cthe_1104	125713844	prepilin-type cleavage/methylation	19233.2	183	21	65	
21	Cthe_1862	125974366	ABC transporter related protein	42056.4	317	31	38	
22	Cthe_1754	125714483	solute-binding protein	35734.5	143	19	48	1
23	Cthe_2709	125975191	hypothetical protein	55140	95	14	19	
24	Cthe_1020	125973535	solute-binding protein	49976.2	385	32	47	
25	Cthe_1754	125714483	solute-binding protein	35734.5	241	29	64	1
26	Cthe_1555	125974064	ABC-type metal ion transport system periplasmic component	32242.5	73	12	32	1
27	Cthe_1869	125714598	ornithine carbamoyltransferase	34235.9	304	20	47	
28	Cthe_1104	125713844	prepilin-type cleavage/methylation	19233.2	539	21	68	

Note:

^*a *^Spots identification numbers (Spots ID) correspond to the numbers in Figure 1.

^*b *^Protein annotations are based on the genome annotation of *C. thermocellum *ATCC 27405.

^*c *^Mr, molecular mass.

**Table 2:** Putative membrane protein complexes of *C. thermocellum*

Complex	**Spots Id**^***d***^	Gene locus	**Protein descriptions**^***e***^	**Approximate mass**^***f ***^**(kDa)**
C1	9	Cthe_2267	V-type ATP synthase subunit A	300
	10	Cthe_2268	V-type ATP synthase beta chain	
C2	10	Cthe_2608	ATP synthase F1, beta subunit	300
	11	Cthe_2606	ATP synthase F1, alpha subunit	
C3	1	Cthe_0423	bifunctional acetaldehyde/alcohol dehydrogenase	>880
C4	15	Cthe_0699	carboxyl transferase	220
C5	19	Cthe_1840	cysteine synthase	130
C6	27	Cthe_1869	ornithine carbamoyltransferase	100
C7	3	Cthe_2253	ATP-dependent metalloprotease FtsH	>880
C8	13	Cthe_0418	polynucleotide phosphorylase/polyadenylase	150
C9	7	Cthe_0016	Ferritin and Dps	440
C10	14	Cthe_3148	ABC transporter related protein	140
C11	16	Cthe_1020	solute-binding protein	190
	18	Cthe_1018	binding-protein-dependent transport systems inner membrane component	
	21	Cthe_1862	ABC transporter related protein	
C12	17	Cthe_1557	ABC transporter related protein ATP-binding protein	140
C13	22	Cthe_1754	solute-binding protein	170
C14	12	Cthe_2348	S-layer-like region; Ig-related	140
C15	20	Cthe_1104	prepilin-type cleavage/methylation	20~180

Note:

^*d *^Spots identification numbers (Spots ID) correspond to the numbers in Figure 1.

^*e *^Protein annotations are based on the genome annotation of *C. thermocellum *ATCC 27405.

^*f *^Approximate mass observed on BN-PAGE.

### Complexes in energy production and conversion

In prokaryotes, three evolutionarily related sub types of ATPases/synthases were found, categorized as F- (F_1_-F_0_-), V- (V_1_-V_0_) and A- (A_1_-A_0_) type ATPases on the basis of their function and taxonomic origins. Although eukaryotes contain both F- and V-ATPases, each highly specialized in its physiological functions; archaea and eubacteria typically contain only one subtype of ATPase [15]. Most eubacteria contain F-ATPases, but some eubacteria contain both F- and V-ATPases, whereas all known archaea contain complexes that are evolutionarily closer to V-ATPases and are referred to as A-ATPases due to their archael origin.

Generally, the F_1_-F_0_-ATP synthase contains eight subunits arranged in two subcomplexes: F_1 _(α_3_, β_3_, γ, δ, ε) and F_0 _(a, b_2_, c_10-14_) [16]. The V_1_-V_0_-ATP synthase contains nine subunits arranged in two subcomplexes: V_1 _(A_3_, B_3_, D, F) and V_0 _(G, E, C, I, L) [17]. Interestingly, in the genome of *C. thermocellum*, there are two ATPase gene clusters: a F_1_-F_0_-ATP synthase (Cthe_2602--Cthe_2609) and V_1_-V_0_-ATP synthase (Cthe_2261-Cthe_2269), both with a complete set of subunits.

We detected two subunits of F_1_-F_0_-ATPase, F_1 _subunit α (Cthe_2606, 55.8 kDa) and F_1 _subunit β (Cthe_2608, 51 kDa), with an estimated molecular mass of 300 kDa and two subunits of V_1_-V_0_-ATPase, V_1 _subunit A (Cthe_2267, 65 kDa) and V_1 _subunit B (Cthe_2268, 50 kDa), with an estimated molecular mass of 300 kDa. These may represent a subcomplex of α_3_β_3 _and A_3_B_3 _in F1 and V_1_, respectively. We conducted a large scale search of ATPase in published genomes of eubacteria from NCBI, 700 genomes were found to contain genes encoding F-type ATPases, 93 genomes contain genes encoding V-type ATPases, and only 44 genomes contain both F-type and V-type ATPases (see Additional file 1). The co-presence of both ATPases in a bacterium is limited to a few genera, which include several *Streptococcus*, *Clostridium*, *Anaeromyxobacter *strains, two *Cyanothece *species, an *Enterococcus faecalis *and a *Nitrosococcus oceani*. We deduce these may reflect unusual ATP generating mechanisms in these bacteria. In this work, we found that both the F- and V-type ATPases are expressed *C. themocellum*. Co-presence of V- and F-type ATPases in a bacterium is uncommon. Previously, only *Enterococcus hirae *was reported to utilize both types of ATPases [18]. The *E. hirae *V-type ATPase differs from typical V-type ATPase in preferentially transporting Na^+ ^[19,20] instead of H^+^. In the thermophilic *Clostridium fervidus*, a second example of Na^+^-pumping V-type ATPase was reported [21]. It is reasonable to speculate that the V-type ATPase in *C. thermocellum *is a Na^+^-pumping ATPase. Most bacteria contain either F-type or V-type ATPase, among those that contain both types of ATPases, new functional variants of ATPases could be identified and their roles in bacterial physiology could be investigated.

Bifunctional acetaldehyde/alcohol dehydrogenase (ALDH-ADH, Cthe_0423, 96 kDa) was detected at over 880 kDa. ADHs could be classified into 3 classes based on their length: short chain ADH (approximately 250 residues) and medium chain ADH (approximately 370 residues) exist in a homotetramer form [22], but a structure of long chain ADH (over 380 amino acids and often as many as 900 amino acid residues) was not reported. The ALDH-ADH of *C. thermocellum *appears to be a long chain ADH and forms a homo-multimer like the ADH in *Entamoeba histolytica *[23]. Alcohol dehydrogenases were reported to be membrane-bound protein complexes [24-26], it is reasonable to observe ADH in *C. thermocellum *membrane fraction.

### Complexes in lipid transport and metabolism

Carboxyl transferase (CT, Cthe_0699, 56 kDa) was identified at ~220 kDa. In eubacteria, CT is part of acetyl coenzyme A carboxylase (ACC) complex, which normally consists of biotin carboxylase (BC), biotin carboxyl carrier protein (BCCP), and CT. Typically, CT contains two subunits in a stable α_2_β_2 _form [27,28]. But, in *Streptomyces coelicolor*, the ACC enzyme has a subunit (590 residues) with fused BC and BCCP domains, and another subunit (530 residues) that contains the fused CT domains [29]. In archaea, ACC is a multi-subunit enzyme, with BC, BCCP and CT subunits. The archael CT subunit is also a single protein (520 residues) in a CT_4 _form, rather than two separate subunits, which is similar to the β subunit (CT) of the ACC from *Streptomyces *[30]. In *C. thermocellum*, CT is a 56 kDa protein, which contains two domains of carboxyl transferase, and we did not detect other ACC subunits on BN/SDS-PAGE. So the CT appears to be a sub complex of CT_4 _not associated with BC and BCCP. CT was also detected at over 880 kDa, which maybe due to precipitation during electrophoresis or CT formed a large complex with other subunits of ACC. Previous studies also suggested ACC may form a membrane-associated protein complex [31,32].

### Complexes in amino acid transport and metabolism

Serine-Acetyl-Transferase (SAT, Cthe_1840, 33.4 kDa), a subunit of cysteine synthase (CS), was detected at ~130 kDa corresponding to the size of intact CS complex. Typical CS complex is composed of one SAT and two O-Acetyl-Serine-(Thiol)-Lyases (OAS-TL, Cthe_1842, 46.5 kDa) [33,34], but we did not detect OAS-TL. It is likely that OAS-TL was masked by the very abundant protein, Cthe_1020. Detection of CS in the membrane fractions has been reported in other studies [9,35].

Ornithine carbamoyltransferase (OTCase, Cthe_1869, 34 kDa) was identified at ~100 kDa, probably in a typical homo-trimer form [36-39]. Some studies suggest that OTCase is a cell surface protein [40,41] whereas Shi *et al. *[42] reported that OTCase maybe a membrane-associated protein based on sequence analyses. Our results support the membrane location of OTCase.

ATP-dependent metalloprotease FtsH (Cthe_2253, 66.6 kDa) was detected at over 880 kDa. FtsH is a cytoplasmic membrane-integrated protein that functions to processively degrade both cytoplasmic and membrane proteins in concert with protein unfolding and is known to form a large membrane-spanning holoenzyme of more than 1000 kDa with the prohibitin-like proteins HflK and HflC [43] or in a hexameric ring structure [44,45]. Although HflK and HflC homologues were not detected from the gel, our results indicate that FtsH forms a large complex on the membrane.

### Complexes in translation, ribosomal structure and biogenesis

Polyribonucleotide phosphorylase (PNPase, Cthe_0418, 77 kDa) was identified at ~150 kDa in the gel at a size of a dimer. It was reported to form a homo-trimer in eukaryotes, bacteria, and archaea [46-50] and was found in membrane fractions [51,52].

### Complexes in inorganic ion transport and metabolism

We detected ferritin (Cthe_0016, 18.6 kDa) at ~440 kDa, indicating that it is intact in a typical 24 mer form on BN-PAGE [53,54]. But ferritin was also detected at over 110 kDa on SDS-PAGE, maybe due to incomplete denaturation. Ferritin is a well known membrane-bound protein.

### Membrane Transport Complexes

Three solute binding proteins (BP, Cthe_1020, Cthe_1555, Cthe_1754), two ATP binding cassette proteins (ABC, Cthe_1557, Cthe_1862), one integral membrane component (IM, Cthe_1018), and an ABC transporter (Cthe_3148) with fused ABC and IM domains were identified from the SDS gel.

ABC transporter diverged into three main classes: Class 1 is comprised of fused ABC and IM domains; Class 2 is comprised of two tandem repeated ABC domains with no IM domains, this class likely does not function as transporters; Class 3 contains independent IM and ABC domains, that correspond to most BP-dependent importers[55]. A typical class 3 ABC transporter complex consists of one BP, two ABCs and two IMs, but the interactions of BP with the complex are weak, so most often only ABC and IM were isolated in a transporter complex [56,57]. In Gram-positive bacteria, BP is either tethered to the cell surface via an N-terminal Cys residue covalently attached to the lipid membrane or by interaction with the IM component of a transporter complex [55].

An ABC transporter (Cthe_3148, 70 kDa) was detected at ~140 kDa, it is a Class 1 ABC transporter with fused ABC and IM domains. The estimated size of Cthe_3148 indicates that it was isolated in an intact dimeric form.

The solute binding protein (Cthe_1020, 49 kDa), the integral membrane protein (Cthe_1018, 32 kDa) and the ATP binding cassette protein (Cthe_1862, 42 kDa) were identified on a vertical line at ~190 kDa. In the genome of *C. thermocellum*, no ATP binding cassette proteins are found near Cthe_1020 and Cthe_1018, and Cthe_1862 is not adjacent to other BP or IM proteins. The identification of these proteins on a vertical line strongly suggests that they form a transporter complex. Cthe_1020 is an abundantly expressed protein under our culture condition, it was detected at ~100 kDa to over 880 kDa, and the high molecular weight spots maybe result of protein precipitation during electrophoresis.

Cthe_1555, Cthe_1556 and Cthe_1557 form an ABC transporter gene cluster in the genome. The ATP binding cassette protein (Cthe_1557, 30 kDa) was detected at an estimated molecular mass of ~140 kDa. But the integral membrane protein Cthe_1556 (26 kDa) and solute binding protein Cthe_1555 (32 kDa) were not detected. The estimated size of this ABC transporter complex suggests it contains two subunits of Cthe_1557, two subunits Cthe_1556 and one subunit of Cthe_1555 as an intact complex. Cthe_1555 was detected at ~100 kDa on a horizontal line with Cthe_1557, which could be due to dissociation of the transporter complex during electrophoresis.

Cthe_1752, Cthe_1753 and Cthe_1754 form an ABC transporter gene cluster in the genome. The solute binding protein (Cthe_1754, 36 kDa) was detected at ~170 kDa. But the integral membrane protein Cthe_1753 (37 kDa) and ATP binding cassette protein Cthe_1752 (30 kDa) was not detected. The size of ABC transporter complex estimated by BN-PAGE, suggests it contains two subunits Cthe_1752, two subunits Cthe_1753 and one subunit of Cthe_1754.

In this study, we did not detect the proteins in other ABC transporter gene clusters studied *in vitro *by Nataf [58] except Cthe_1020.

### Other protein complexes

In Gram-positive bacteria, S-layer proteins are known to non-covalently attach to the pyruvylated negatively-charged secondary cell wall polymers (SCWP) by the surface layer homology (SLH) domains [59-61]. We detected S-layer protein (Cthe_2348, 113 kDa) at ~140 kDa, probably in a monomeric form, and there maybe a fragment of SCWP tethered with S-layer protein.

Prepilin (Cthe_1104, 19 kDa) was identified from 20 kDa to 180 kDa in the SDS gel, this may reflect that the prepilins were in a process of pilin assembly [62].

### Hypothetical proteins

Three hypothetical proteins (Cthe_0858, Cthe_2693 and Cthe_2709) were detected in our membrane sample. Although Cthe_0858 showed weak similarity to domains designated PRK 13665, pfam 12127 and COG4864. The functions of these domains or their corresponding proteins are not known.

## Discussions on the method

Previously, a Bicine-dSDS-PAGE method was developed to separate membrane proteins [63], it employs strong solubilization conditions in both dimensions of SDS-PAGE. The method is suitable for membrane proteomics study, and was used to identify 81 membrane proteins from *C. thermocellum *[64]. In this work, BN/SDS-PAGE was applied in the analysis of membrane protein complexes of *C. thermocellum *for the first time. Although the first dimensional BN-PAGE was carefully optimized, the second dimensional SDS-PAGE proved difficult to perform probably because the solubilization factors were altered during SDS electrophoresis. So technically, it is still a huge challenge to isolate and solubilize membrane protein complexes as well as to separate these complexes on BN/SDS-PAGE. To isolate intact protein complexes, gentle cell disruption method must be considered. We used sonication conditions (with low sonication power and long sonication intervals), that sufficiently protected complex stability. After repeat optimization of various conditions, we were able to solubilize and separate a sub-fraction of membrane protein complexes and to identify 24 membranes proteins representing 13 intact or sub protein complexes. Most of the proteins identified were previously reported to be membrane proteins, thus validating our sample preparation protocol. Many protein complexes we reported were identified for the first time in *C. thermocellum*, thus our findings and protocol paved the way for future detailed characterization of these complexes. BN/SDS-PAGE is a suitable approach for large scale protein-protein interaction investigation, and it is probably the only method of choice to analyze membrane protein complexes on proteomic scale. This method allowed us to detect the simultaneous expression of two sets of ATP synthases (V- and F-type ATPases) in *C. thermocellum*, and this finding provides strong bases for the future investigation into the distinct roles of these ATPases in this bacterium.

## Conclusions

Two dimensional blue native/SDS-PAGE was used to detect membrane protein complexes in *C. thermocellum *and revealed the simultaneous expression of two sets of ATP synthases. The protocol developed in this work paves the way for further functional characterization of membrane protein complexes in this bacterium.

## Methods

### Bacterial strains and growth conditions

*C. thermocellum *DSM 1237 (ATCC 27405) was obtained from Deutsche Sammlung von Mikroorganismen und Zellkulturen. It was cultured at 60°C in a medium containing: (NH4)_2_SO_4 _1.30 g, MgCl_2_·6H_2_O, 2.60 g, KH_2_PO_4 _1.43 g, K_2_HPO_4_·3H_2_O 7.20 g, CaCl_2_·2H_2_O 0.13 g, Na-β-glycerophosphate 6.00 g, FeSO_4_·7H_2_O 1.10 mg, Glutathione 0.25 g, Yeast Extract 4.50 g, Resazurin 1.00 mg, Cellobiose 5.00 g per litre water. The basal medium was adjusted to pH 7.2 with 10% NaOH and the headspace of the medium container was continuously flushed with oxygen-free nitrogen. All chemicals were purchased from Sigma-Aldrich (St. Louis, MO, USA) unless otherwise noted.

### Preparation of crude membrane protein fraction

*C. thermocellum *cells were harvested at late log phase by centrifugation at 8000 g for 10 min at 4°C, washed twice with 50 mM Tris-HCl (pH 7.5), and then re-suspended in 50 mM Tris-HCl (pH 7.5) containing 0.5 mM PMSF (Amresco). The re-suspended cells were disrupted by gentle sonication on ice (5 s pulse of sonication with 10 s intervals for 12 min) and centrifuged at 20,000 g for 30 min at 4°C. The pellet was discarded and the supernatant was centrifuged at 200,000 g for 60 min to obtain the membrane fraction. The membrane fraction was washed twice and finally re-suspended in solubilization buffer (50 mM NaCl, 50 mM Imidazole/HCl, 2 mM 6-Aminohexanoic acid (ACA), 1 mM EDTA, pH 7.0) and further treated for BN gel or stored at -80°C. Protein concentration was determined using the Bradford assay [65].

Protein complexes were solubilized at 4°C in solubilization buffer containing varying amounts of detergents. Triton X-100, DDM, Sulfobetaine SB10 and 3-[(3-cholamidopropyl) dimethylamonio]-1-propanesulfonate (Chaps) at concentrations ranging from 0.5% to 2.0% (w/v) were tested. Solubilization with 1.0% (w/v) DDM was found to be most effective, as evidenced by the number of complexes in the BN gel, the intensity and the molecular mass range of these complexes. Subsequent experiments were therefore performed using 1.0% (w/v) DDM as detergent. Following solubilization, samples were cleared by centrifugation at 200,000 g for 30 min at 4°C. The supernatant was mixed with 15 μl of G250 solution (5% (w/v) SERVA Blue G (SERVA Electrophoresis GmbH) in 500 mM ACA buffer) and loaded onto the BN gel.

### Two dimensional BN/SDS PAGE

BN-PAGE and SDS-PAGE were performed using a DYY-23A apparatus (product of Beijing WoDeLife Sciences Instrument Company). In the first dimensional BN-PAGE, approximately 40 μg of protein was loaded. A 3.5% stacking and a 4-15% separating gel (gel dimensions 10 cm×10 cm×1.5 mm) were used. Buffers and gel compositions used were the same as described by Wittig et al [66]. Electrophoresis was conducted at 100 V for 30 min, and following electrophoresis was performed with the current limited to 15 mA and voltage limited to 300 V. Ferritin, catalase and BSA from Amersham Biosciences (Sweden) were used as markers to indicate the sizes of 880, 440, 250, 132 and 66 kDa. BN-polyacrylamide gel strips were cut from the first dementional gel for use in the second dimensional SDS-PAGE.

For the second dimensional SDS-PAGE, strips of the first dimensional BN-PAGE were cut and soaked in 5% (w/v) SDS, 1% (w/v) 2-Mercaptoethanol for 2 h. SDS-PAGEs were performed using a 4% stacking and a 12% separating gel according to standard protocols. Gels were fixed in 50% (v/v) methanol and 12% (v/v) acetic acid for 1 hour and then stained with 0.25% (w/v) Coomassie Blue R250 in 10% (v/v) acetic acid and 50% (v/v) methanol. A series of proteins (Tiangen Company, China) with the sizes of 116, 66.2, 45, 35, 25, 18.4 and 14.4 kDa were used as markers.

### MALDI-TOF MS and MS/MS

Protein spots from SDS gel were excised manually. In-gel trypsin digestion was carried out as previously described [67]. A 0.4 μl aliquot of the concentrated tryptic peptide mixture in 0.1% trifluoroacetic acid (TFA) was mixed with 0.4 μl of α-cyano-4-hydroxycinnamic acid (CHCA) matrix solution (5 mg/ml CHCA in 50% ACN/0.1% TFA) and spotted onto a freshly cleaned target plate. After air drying, the crystallized spots were analyzed on the Applied Biosystems 4700 Proteomics Analyzer MALDI-TOF/TOF (Applied Biosystems, Framingham, MA, USA). MS calibration was automatically performed by a peptide standard Kit (Applied Biosystems) containing des-Arg1-bradykinin (m/z 904), Angiotensin I (m/z 1296.6851), Glu1-fibrinopeptide B (m/z 1570.6774), Adrenocorticotropic hormone (ACTH) (1-17, m/z 2903.0867), ACTH (18-39, m/z 2465.1989), and ACTH (7-38, m/z 3657.9294) and MS/MS calibration was performed by the MS/MS fragment peaks of Glu1-fibrinopeptide B. All MS mass spectra were recorded in the reflector positive mode using a laser operated at a 200 Hz repetition rate with wavelength of 355 nm. The accelerated voltage was operated at 2 kV. The MS/MS mass spectra were acquired by the data dependent acquisition method with the 10 strongest precursors selected from one MS scan. All MS and MS/MS spectra were obtained by accumulation of at least 1000 and 3000 laser shots, respectively. Neither baseline subtraction nor smoothing was applied to recorded spectra. MS and MS/MS data were analyzed and peak lists were generated using GPS Explorer 3.5 (Applied Biosystems). MS peaks were selected between 700 and 3500 Da and filtered with a signal to noise ratio greater than 20. A peak intensity filter was used with no more than 50 peaks per 200 Da. MS/MS peaks were selected based on a signal to noise ratio greater than 10 over a mass range of 60 Da to 20 Da below the precursor mass. MS and MS/MS data were analyzed using MASCOT™ 2.0 search engine (Matrix Science, London, UK) to search against the *C. themocellum *protein sequence database downloaded from NCBI database on December 01 2008. Searching parameters were as follows: trypsin digestion with one missed cleavage, variable modifications (oxidation of methionine and carbamidomethylation of cysteine), and the mass tolerance of precursor ion and fragment ion at 0.2 Da for +1 charged ions. For all proteins successfully identified by Peptide Mass Fingerprint and/or MS/MS, Mascot score greater than 53 (the default MASCOT threshold for such searches) was accepted as significant (*p *value < 0.05). The false positive rate was estimated based on reverse database search. The false positive rate = peptide fragment numbers detected in reverse database search/(peptide fragment numbers in forward database search+ peptide fragment numbers in reverse database search) × 100%.

## Authors' contributions

YP performed all experiments and wrote the manuscript. YL performed the MALDI-TOF and wrote the MALDI-TOF MS and MS/MS part of the manuscript. TY and KF were involved in study design and revising the manuscript. YZ performed the database search of ATPase in bacteria. KY supervised the project and revised the manuscript. All authors read and approved the final manuscript.

## Supplementary Material

Additional file 1 **Results of ATPase search in published genomes of eubacteria from NCBI**. Table listing the eubacteria which contain F-type ATPase, V-type ATPase or both F-type and V-type ATPases. Click here for file
