# Supplementary material for: A Blue Native-PAGE analysis of membrane protein complexes in Clostridium thermocellum
Source: BMC Microbiol. 2011 Jan 26;11:22. doi: 10.1186/1471-2180-11-22 (PMC3039559; doi:10.1186/1471-2180-11-22)
Supplement: Additional file 1 — Results of ATPase search in published genomes of eubacteria from NCBI. Table listing the eubacteria which contain F-type ATPase, V-type ATPase or both F-type and V-type ATPases. [file 1471-2180-11-22-S1.PDF]

Supplementary data Table 1. Results of ATPase search in published genomes of eubacteria from NCBI

Bacteria containing F-type ATPase	Bacteria containing V-type ATPase	Bacteria containing both F-type and V-type ATPases
Acinetobacter baumannii AB307-0294	*Anaeromyxobacter* sp. Fw109-5	*Anaeromyxobacter* sp. Fw109-5
Alkalilimnicola ehrlichii MLHE-1	*Anaeromyxobacter dehalogenans* 2CP-1	*Anaeromyxobacter dehalogenans* 2CP-1
Anaplasma marginale str. Florida	*Anaeromyxobacter* sp. K	*Anaeromyxobacter* sp. K
Bacillus cereus AH187	*Anaeromyxobacter dehalogenans* 2CP-C	*Anaeromyxobacter dehalogenans* 2CP-C
Azoarcus sp. BH72	*Borrelia burgdorferi* ZS7	Clostridium *perfringens* str. 13
Bacillus anthracis str. Sterne	*Borrelia recurrentis* A1	*Cyanothece* sp. PCC 8801
Bacillus cereus AH820	*Borrelia turicatae* 91E135	*Clostridium botulinum* Ba4 str. 657
Acinetobacter baumannii AYE	*Brachyspira hyodysenteriae* WA1	*Clostridium botulinum* A3 str. Loch Maree
Aggregatibacter aphrophilus NJ8700	*Bacteroides fragilis* NCTC 9343	*Clostridium botulinum* F str. Langeland
Arcobacter butzleri RM4018	*Bacteroides vulgatus* ATCC 8482	*Clostridium botulinum* A str. Hall
Bacillus anthracis str. A0248	*Bacteroides fragilis* YCH46	*Clostridium botulinum* B1 str. Okra
Aggregatibacter actinomycetemcomitans D11S-1	*Bacteroides thetaiotaomicron* VPI-5482	*Clostridium novyi* NT
Arthrobacter aurescens TC1	*Borrelia duttonii* Ly	*Cyanothece* sp. PCC 8802
Aromatoleum aromaticum EbN1	*Borrelia burgdorferi* B31	*Clostridium botulinum* E3 str. Alaska E43
Azotobacter vinelandii DJ	*Borrelia afzelii* PKo	*Clostridium botulinum* B str. Eklund 17B
Acinetobacter baumannii ATCC 17978	*Borrelia hermsii* DAH	*Clostridium perfringens* SM101
Acidothermus cellulolyticus 11B	*Chlamydophila pneumoniae* J138	*Clostridium thermocellum* ATCC 27405
Acidovorax citrulli AAC00-1	*Clostridium botulinum* A str. Hall	*Clostridium botulinum* A2 str. Kyoto
Anaeromyxobacter dehalogenans 2CP-1	*Clostridium tetani* E88	*Clostridium perfringens* ATCC 13124
Azorhizobium caulinodans ORS 571	*Clostridium botulinum* A3 str. Loch Maree	*Clostridium botulinum* A str. ATCC 3502
Anaeromyxobacter dehalogenans 2CP-C	*Clostridium botulinum* Ba4 str. 657	*Clostridium botulinum* A str. ATCC 19397
Aeromonas hydrophila subsp. *hydrophila* ATCC 7966	*Chlamydia trachomatis* L2b/UCH-1/*proctitis*	*Enterococcus faecalis* V583
Alteromonas macleodii 'Deep ecotype'	*Cyanothece* sp. PCC 8801	*Nitrosococcus oceani* ATCC 19707
Anabaena variabilis ATCC 29413	*Desulfohalobium retbaense* DSM 5692	*Streptococcus pneumoniae* ATCC 700669
Acidovorax ebreus TPSY	*Chlamydophila pneumoniae* AR39	*Streptococcus pyogenes* MGAS9429
Bacillus cereus B4264	*Clostridium perfringens* str. 13	*Streptococcus pyogenes* MGAS315

Arthrobacter chlorophenolicus A6	*Clostridium botulinum* F str. Langeland	*Streptococcus pyogenes* MGAS10394
Actinobacillus pleuropneumoniae L20	*Clostridium botulinum* B1 str. Okra	*Streptococcus pneumoniae* TIGR4
Actinobacillus succinogenes 130Z	*Chlamydophila felis* Fe/C-56	*Streptococcus pneumoniae* G54
Anaerocellum thermophilum DSM 6725	*Clostridium phytofermentans* ISDg	*Streptococcus pneumoniae* JJA
Bacillus anthracis str. CDC 684	*Chlamydophila pneumoniae* CWL029	*Streptococcus pyogenes* str. Manfredo
Aeromonas salmonicida subsp. *salmonicida* A449	*Clostridium novyi* NT	*Streptococcus pyogenes* NZ131
Acinetobacter baumannii ACICU	*Cyanothece* sp. PCC 8802	*Streptococcus pyogenes* MGAS2096
Actinobacillus pleuropneumoniae serovar 7 str. AP76	*Clostridium difficile* 630	*Streptococcus pyogenes* M1 GAS
Bacillus anthracis str. Ames	*Chlamydia trachomatis* B/Jali20/OT	*Streptococcus gordonii* str. Challis substr. CH1
Bacillus anthracis str. 'Ames Ancestor'	*Clostridium botulinum* E3 str. Alaska E43	*Streptococcus pyogenes* MGAS5005
Bacillus cereus ATCC 14579	*Chlamydia trachomatis* A/HAR-13	*Streptococcus pyogenes* MGAS10270
Bacillus amyloliquefaciens FZB42	*Clostridium botulinum* B str. Eklund 17B	*Streptococcus pyogenes* MGAS8232
Anaplasma marginale str. *St*. Maries	*Deinococcus deserti* VCD115	*Streptococcus pyogenes* SSI-1
Anaeromyxobacter sp. Fw109-5	*Clostridium perfringens* SM101	*Streptococcus sanguinis* SK36
Alcanivorax borkumensis SK2	*Chlamydophila caviae* GPIC	*Streptococcus pyogenes* MGAS6180
Acinetobacter baumannii SDF	*Chlamydia trachomatis* B/TZ1A828/OT	*Streptococcus pneumoniae* Hungary19A-6
Acinetobacter sp. ADP1	*Chlamydia trachomatis* D/UW-3/CX	*Streptococcus pyogenes* MGAS10750
Actinobacillus pleuropneumoniae serovar 3 str. JL03	*Clostridium difficile* CD196	*Streptococcus pneumoniae* CGSP14
Bacillus cereus ATCC 10987	*Clostridium thermocellum* ATCC 27405	
Anaeromyxobacter sp. K	*Chlamydia muridarum* Nigg	
Bacillus cereus 03BB102	*Chlamydophila abortus* S26/3	
Anaplasma phagocytophilum HZ	*Chlamydophila pneumoniae* TW-183	
Acaryochloris marina MBIC11017	*Clostridium botulinum* A2 str. Kyoto	
Acetobacter pasteurianus IFO 3283-01	*Candidatus Protochlamydia amoebophila* UWE25	
Acidovorax sp. JS42	*Clostridium difficile* R20291	
Acinetobacter sp. DR1	*Deinococcus geothermalis* DSM 11300	
Acidiphilium cryptum JF-5	*Clostridium perfringens* ATCC 13124	
Anoxybacillus flavithermus WK1	*Clostridium botulinum* A str. ATCC 3502	

Acinetobacter baumannii AB0057<br>*Bacillus thuringiensis serovar konkukian* str. 97-27<br>*Baumannia cicadellinicola* str. Hc (*Homalodisca coagulata*)<br>*Bradyrhizobium* sp. BTAi1<br>*Bacillus cereus* Q1<br>*Bradyrhizobium* sp. ORS278<br>*Bartonella grahamii* as4aup<br>*Campylobacter jejuni* subsp. *jejuni* 81116<br>*Bartonella tribocorum* CIP 105476<br>*Buchnera aphidicola* str. Sg (*Schizaphis graminum*)<br>*Candidatus Accumulibacter phosphatis clade* IIA str. UW-1<br>*Bacillus licheniformis* ATCC 14580<br>*Bacillus cereus* G9842<br>*Buchnera aphidicola* str. Bp (*Baizongia pistaciae*)<br>*Bradyrhizobium japonicum* USDA 110<br>*Bartonella henselae* str. Houston-1<br>*Candidatus Blochmannia pennsylvanicus* str. BPEN<br>*Bifidobacterium animalis* subsp. *lactis* Bl-04<br>*Buchnera aphidicola* str. APS (Acyrthosiphon pisum)<br>*Bifidobacterium animalis* subsp. *lactis* DSM 10140<br>*Campylobacter jejuni* subsp. *jejuni* NCTC 11168<br>*Bifidobacterium longum* subsp. *infantis* ATCC 15697<br>*Bacillus cytotoxicus* NVH 391-98<br>*Campylobacter jejuni* subsp. *doylei* 269.97<br>*Bifidobacterium longum* DJO10A<br>*Campylobacter hominis* ATCC BAA-381	*Clostridium botulinum* A str. ATCC 19397<br>*Chlamydia trachomatis* 434/Bu<br>*Fusobacterium nucleatum* subsp. *nucleatum* ATCC 25586<br>*Enterococcus faecalis* V583<br>*Finegoldia magna* ATCC 29328<br>*Fibrobacter succinogenes* subsp. *succinogenes* S85<br>*Halothermothrix orenii* H 168<br>*Porphyromonas gingivalis* ATCC 33277<br>*Nitrosococcus oceani* ATCC 19707<br>*Parabacteroides distasonis* ATCC 8503<br>*Porphyromonas gingivalis* W83<br>*Streptococcus pneumoniae* ATCC 700669<br>*Streptococcus pyogenes* MGAS9429<br>*Streptococcus pyogenes* MGAS315<br>*Streptococcus pyogenes* MGAS10394<br>*Streptococcus pneumoniae* TIGR4<br>*Streptococcus pneumoniae* G54<br>*Streptococcus pneumoniae* JJA<br>*Streptococcus pyogenes* str. Manfredo<br>*Streptococcus pyogenes* NZ131<br>*Streptococcus pyogenes* MGAS2096<br>*Streptococcus pyogenes* M1 GAS<br>*Streptococcus gordonii* str. Challis substr. CH1<br>*Streptococcus pyogenes* MGAS5005<br>*Streptococcus pyogenes* MGAS10270<br>*Streptococcus pyogenes* MGAS8232<br>*Streptococcus pyogenes* SSI-1	

Buchnera aphidicola str. 5A (*Acyrthosiphon* pisum)	*Streptococcus sanguinis* SK36	
Bacillus subtilis subsp. *subtilis* str. 168	*Streptococcus pyogenes* MGAS6180	
Bacillus clausii KSM-K16	*Streptococcus pneumoniae* Hungary19A-6	
Bartonella quintana str. Toulouse	*Streptococcus pyogenes* MGAS10750	
Beijerinckia indica subsp. *indica* ATCC 9039	*Streptococcus pneumoniae* CGSP14	
Campylobacter fetus subsp. *fetus* 82-40	*Treponema denticola* ATCC 35405	
Bifidobacterium adolescentis ATCC 15703	*Treponema pallidum* subsp. *pallidum* SS14	
Bacillus weihenstephanensis KBAB4	*Treponema pallidum* subsp. *pallidum* str. Nichols	
Bartonella bacilliformis KC583	*Thermus thermophilus* HB27	
Campylobacter curvus 525.92	*Thermus thermophilus* HB8	
Campylobacter jejuni subsp. *jejuni* 81-176	*Deinococcus radiodurans* R1	
Candidatus Blochmannia floridanus	*Borrelia garinii* PBi	
Bordetella petrii DSM 12804		
Campylobacter lari RM2100		
Bifidobacterium animalis subsp. lactis AD011		
Bacillus thuringiensis BMB171		
Campylobacter concisus 13826		
Bacillus cereus E33L		
Bacillus thuringiensis str. Al *Hakam*		
Bacillus halodurans C-125		
Bordetella avium 197N		
Bifidobacterium longum NCC2705		
Campylobacter jejuni RM1221		
Buchnera aphidicola str. Tuc7 (*Acyrthosiphon pisum*)		
Bacillus pumilus SAFR-032		
Caldicellulosiruptor saccharolyticus DSM 8903		
Chlorobium luteolum DSM 273		
Chlorobaculum parvum NCIB *8327*		

Coxiella burnetii RSA 331<br>*Chlorobium phaeobacteroides* DSM 266<br>*Clostridium beijerinckii* NCIMB 8052<br>*Clostridium botulinum* A3 str. Loch Maree<br>*Corynebacterium jeikeium* K411<br>*Chlorobium chlorochromatii* CaD3<br>*Clostridium cellulolyticum* H10<br>*Candidatus Liberibacter asiaticus* str. psy62<br>*Corynebacterium urealyticum* DSM 7109<br>*Clostridium novyi* NT<br>*Candidatus Vesicomyosocius okutanii* HA<br>*Chromohalobacter salexigens* DSM 3043<br>*Candidatus* Ruthia *magnifica* str. Cm (*Calyptogena magnifica*)<br>*Clostridium botulinum* B str. *Eklund* 17B<br>*Clostridium acetobutylicum* ATCC 824<br>*Clostridium perfringens* SM101<br>*Cellvibrio japonicus* Ueda107<br>*Candidatus Pelagibacter ubique* HTCC1062<br>*Clostridium botulinum* A2 str. Kyoto<br>*Colwellia psychrerythraea* 34H<br>*Corynebacterium glutamicum* R<br>*Chromobacterium violaceum* ATCC 12472<br>*Clostridium botulinum* A str. ATCC 19397<br>*Clostridium botulinum* A str. Hall<br>*Clostridium kluyveri* NBRC 12016<br>*Dichelobacter nodosus* VCS1703A<br>*Coxiella burnetii* Dugway 5J108-111		

Desulfobacterium autotrophicum HRM2<br>*Clostridium botulinum* Ba4 str. 657<br>*Corynebacterium efficiens* YS-314<br>*Cyanothece sp*. PCC 8801<br>*Delftia acidovorans* SPH-1<br>*Clostridium perfringens* str. 13<br>*Clostridium botulinum* F str. Langeland<br>*Cyanothece sp*. PCC 7424<br>*Coxiella burnetii* RSA 493<br>*Clostridium botulinum* B1 str. Okra<br>*Cronobacter turicensis* z3032<br>*Corynebacterium aurimucosum* ATCC 700975<br>*Clavibacter michiganensis* subsp. *sepedonicus*<br>*Desulfovibrio salexigens* DSM 2638<br>*Coxiella burnetii* CbuK_Q154<br>*Cyanothece* sp. PCC 7425<br>*Clavibacter michiganensis* subsp. *michiganensis* NCPPB 382<br>*Chelativorans* sp. BNC1<br>*Cyanothece* sp. PCC 8802<br>*Clostridium botulinum* E3 str. Alaska E43<br>*Caulobacter crescentus* NA1000<br>*Caulobacter crescentus* CB15<br>*Corynebacterium kroppenstedtii* DSM 44385<br>*Caulobacter* sp. K31<br>*Chlorobium phaeovibrioides* DSM 265<br>*Dechloromonas aromatica* RCB<br>*Catenulispora acidiphila* DSM 44928		

Clostridium kluyveri DSM 555<br>*Coxiella burnetii* CbuG_Q212<br>*Corynebacterium glutamicum* ATCC 13032<br>*Citrobacter koseri* ATCC BAA-895<br>*Chlorobium tepidum* TLS<br>*Chlorobium phaeobacteroides* BS1<br>*Clostridium thermocellum* ATCC 27405<br>*Cronobacter sakazakii* ATCC BAA-894<br>*Desulfomicrobium baculatum* DSM 4028<br>*Chloroherpeton thalassium* ATCC 35110<br>*Clostridium perfringens* ATCC 13124<br>*Clostridium botulinum* A str. ATCC 3502<br>*Escherichia coli* O157:H7 str. TW14359<br>*Frankia alni* ACN14a<br>*Geobacillus kaustophilus* HTA426<br>*Escherichia coli* IAI1<br>*Ehrlichia ruminantium* str. Welgevonden<br>*Francisella tularensis* subsp. *mediasiatica* FSC147<br>*Francisella tularensis* subsp. *holarctica* FTNF002-00<br>*Dickeya zeae* Ech1591<br>*Escherichia coli* UTI89<br>*Enterococcus faecalis* V583<br>Geobacillus sp. WCH70<br>*Escherichia coli* ATCC 8739<br>*Escherichia coli* O26:H11 str. 11368<br>*Escherichia coli* IAI39<br>*Ehrlichia canis* str. Jake<br>*Ehrlichia chaffeensis* str. Arkansas		

Escherichia coli 55989<br>*Escherichia coli* 536<br>*Francisella philomiragia* subsp. philomiragia ATCC 25017<br>*Ehrlichia ruminantium* str. Gardel<br>*Escherichia coli* E24377A<br>*Francisella tularensis* subsp. *tularensis* FSC198<br>*Dinoroseobacter shibae* DFL 12<br>*Escherichia coli* UMN026<br>*Escherichia coli* B str. REL606<br>*Escherichia coli* O127:H6 str. E2348/69<br>*Geobacillus* sp. Y412MC61<br>*Enterobacter* sp. 638<br>*Francisella novicida* U112<br>*Enterobacter cloacae* subsp. cloacae ATCC 13047<br>*Escherichia fergusonii* ATCC 35469<br>*Escherichia coli* O157:H7 EDL933<br>*Escherichia coli* SE11<br>*Frankia sp*. EAN1pec<br>*Escherichia coli* O157:H7 str. EC4115<br>*Escherichia coli* str. K-12 substr. DH10B<br>*Francisella tularensis* subsp. tularensis WY96-3418<br>*Escherichia coli* HS<br>*Erwinia pyrifoliae* Ep1/96<br>*Eubacterium rectale* ATCC 33656<br>*Escherichia coli* APEC O1<br>*Escherichia coli* BL21(DE3)<br>*Geobacillus thermodenitrificans* NG80-2		

Francisella tularensis subsp. holarctica OSU18<br>*Escherichia coli* CFT073<br>*Escherichia coli* SMS-3-5<br>*Escherichia coli* O111:H- str. 11128<br>*Escherichia coli* S88<br>*Escherichia coli* O103:H2 str. 12009<br>*Escherichia coli* O157:H7 str. Sakai<br>*Fervidobacterium nodosum* Rt17-B1<br>*Francisella tularensis* subsp. holarctica<br>*Francisella tularensis* subsp. tularensis SCHU S4<br>*Erythrobacter litoralis* HTCC2594<br>*Eubacterium eligens* ATCC 27750<br>*Erwinia tasmaniensis* Et1/99<br>*Escherichia coli* ED1a<br>*Frankia* sp. CcI3<br>*Escherichia coli* BW2952<br>*Helicobacter hepaticus* ATCC 51449<br>*Methylobacterium nodulans* ORS 2060<br>*Mycoplasma arthritidis* 158L3-1<br>*Leptothrix cholodnii* SP-6<br>*Helicobacter pylori* P12<br>*Haemophilus parasuis* SH0165<br>*Methylacidiphilum infernorum* V4<br>*Klebsiella pneumoniae* 342<br>*Leifsonia xyli* subsp. *xyli* str. CTCB07<br>*Gluconacetobacter diazotrophicus* PAl 5<br>*Haemophilus influenzae* Rd KW20<br>*Listeria monocytogenes* 08-5578		

Mycobacterium avium subsp. *paratuberculosis* K-10<br>*Mycobacterium tuberculosis* F11<br>*Klebsiella pneumoniae* NTUH-K2044<br>*Mycobacterium bovis* BCG str. Tokyo 172<br>*Haemophilus somnus* 129PT<br>*Mycobacterium tuberculosis* CDC1551<br>*Lactobacillus salivarius* UCC118<br>*Helicobacter pylori* 26695<br>*Methylobacterium extorquens* AM1<br>*Mycobacterium avium* 104<br>*Haemophilus influenzae* PittGG<br>*Mycobacterium ulcerans* Agy99<br>*Mycobacterium tuberculosis* KZN 1435<br>*Klebsiella pneumoniae* subsp. pneumoniae MGH 78578<br>*Lactobacillus helveticus* DPC 4571<br>*Lactobacillus johnsonii* NCC 533<br>*Helicobacter pylori* B38<br>*Lactococcus lactis* subsp. *cremoris* SK11<br>*Lactobacillus delbrueckii* subsp. *bulgaricus* ATCC BAA-365<br>*Haemophilus influenzae* 86-028NP<br>*Mycoplasma agalactiae* PG2<br>*Haemophilus somnus* 2336<br>*Mycoplasma conjunctivae* HRC/581<br>*Methylococcus capsulatus* str. Bath<br>*Mycoplasma gallisepticum* str. R(low)<br>*Helicobacter acinonychis* str. Sheeba<br>*Mesoplasma florum* L1		

Helicobacter pylori HPAG1<br>*Microcystis aeruginosa* NIES-843<br>*Legionella pneumophila* subsp. *pneumophila* str. Philadelphia 1<br>*Mycobacterium* sp. KMS<br>*Helicobacter* pylori Shi470<br>*Methylobacterium* populi BJ001<br>*Marinobacter aquaeolei* VT8<br>*Laribacter hongkongensis* HLHK9<br>*Halorhodospira halophila* SL1<br>*Methylobacterium extorquens* DM4<br>*Macrococcus caseolyticus* JCSC5402<br>*Mycoplasma capricolum* subsp. *capricolum* ATCC 27343<br>*Idiomarina loihiensis* L2TR<br>*Legionella pneumophila* str. Lens<br>*Listeria monocytogenes* str. 4b F2365<br>*Methylobacillus flagellatus* KT<br>*Methylobacterium chloromethanicum* CM4<br>*Lactobacillus delbrueckii* subsp. *bulgaricus* ATCC 11842<br>*Listeria welshimeri serovar* 6b str. SLCC5334<br>*Micrococcus luteus* NCTC 2665<br>*Lysinibacillus sphaericus* C3-41<br>*Hirschia baltica* ATCC 49814<br>*Jannaschia sp*. CCS1<br>*Janthinobacterium* sp. Marseille<br>*Lactococcus lactis* subsp. *lactis* Il1403<br>*Methylobacterium radiotolerans* JCM 2831<br>*Methylobacterium* sp. 4-46		

Mycobacterium smegmatis str. MC2 155<br>*Gluconobacter oxydans* 621H<br>*Mycobacterium* sp. MCS<br>*Maricaulis maris* MCS10<br>*Magnetococcus* sp. MC-1<br>*Kosmotoga olearia* TBF 19.5.1<br>*Lactobacillus acidophilus* NCFM<br>*Helicobacter pylori* G27<br>*Methylotenera mobilis* JLW8<br>*Listeria monocytogenes* Clip80459<br>*Legionella pneumophila* str. Paris<br>*Mannheimia succiniciproducens* MBEL55E<br>*Mesorhizobium loti* MAFF303099<br>*Helicobacter pylori* J99<br>*Mycobacterium* sp. JLS<br>*Mycobacterium vanbaalenii* PYR-1<br>*Magnetospirillum magneticum* AMB-1<br>*Methylocella silvestris* BL2<br>*Lactococcus lactis* subsp. *cremoris* MG1363<br>*Granulibacter bethesdensis* CGDNIH1<br>*Methylovorus sp*. SIP3-4<br>*Mycobacterium bovis* BCG str. Pasteur 1173P2<br>*Kocuria rhizophila* DC2201<br>*Mycobacterium tuberculosis* H37Ra<br>*Marinomonas sp*. MWYL1<br>*Haemophilus ducreyi* 35000HP<br>*Hahella chejuensis* KCTC 2396<br>*Herminiimonas arsenicoxydans*		

Mycoplasma hyopneumoniae 232<br>*Lactobacillus sakei* subsp. *sakei* 23K<br>*Legionella pneumophila* str. Corby<br>*Mycobacterium gilvum* PYR-GCK<br>*Gloeobacter violaceus* PCC 7421<br>*Haemophilus influenzae* PittEE<br>*Mycobacterium marinum* M<br>*Mycoplasma genitalium* G37<br>*Listeria monocytogenes* 08-5923<br>*Lactobacillus plantarum* JDM1<br>*Hyphomonas neptunium* ATCC 15444<br>*Mycobacterium leprae* Br4923<br>*Methylobacterium extorquens* PA1<br>*Methylibium petroleiphilum* PM1<br>*Oceanobacillus iheyensis* HTE831<br>*Prochlorococcus marinus* str. NATL1A<br>*Psychrobacter* sp. PRwf-1<br>*Rickettsia rickettsii* str. 'Sheila Smith'<br>*Orientia tsutsugamushi* str. Boryong<br>*Pseudomonas aeruginosa* PAO1<br>*Ruegeria sp*. TM1040<br>*Prochlorococcus marinus* str. MIT 9211<br>*Rhizobium etli* CFN 42<br>*Neisseria gonorrhoeae* NCCP11945<br>*Rhodopseudomonas palustris* BisA53<br>*Rickettsia felis* URRWXCal2<br>*Pseudomonas mendocina ymp*<br>*Rickettsia canadensis* str. McKiel		

Pseudomonas fluorescens SBW25<br>*Prochlorococcus marinus* str. MIT 9301<br>*Mycoplasma pulmonis* UAB CTIP<br>*Nitrosospira multiformis* ATCC 25196<br>*Pectobacterium atrosepticum* SCRI1043<br>*Prochlorococcus marinus* str. MIT 9303<br>*Psychrobacter arcticus* 273-4<br>*Pseudomonas stutzeri* A1501<br>*Rickettsia africae* ESF-5<br>*Pseudomonas syringae* pv. *phaseolicola* 1448A<br>*Prochlorococcus marinus* subsp. *pastoris* str. CCMP1986<br>*Rickettsia typhi* str. Wilmington<br>*Rickettsia conorii* str. Malish 7<br>*Mycoplasma mycoides* subsp. *mycoides* SC str. PG1<br>*Neisseria meningitidis* FAM18<br>*Nitrobacter hamburgensis* X14<br>*Prochlorococcus marinus* str. MIT 9515<br>*Neorickettsia risticii* str. Illinois<br>*Pseudomonas fluorescens* Pf-5<br>*Prochlorococcus marinus* str. MIT 9312<br>*Mycoplasma hyopneumoniae* 7448<br>*Rhodospirillum centenum* SW<br>*Pseudomonas putida* KT2440<br>*Mycoplasma synoviae* 53<br>*Rickettsia bellii* OSU 85-389<br>*Prosthecochloris aestuarii* DSM 271<br>*Rhodococcus jostii* RHA1<br>*Prochlorococcus marinus* str. MIT 9215		

Novosphingobium aromaticivorans DSM 12444<br>*Rhizobium leguminosarum* bv. *trifolii* WSM1325<br>*Parvibaculum lavamentivorans* DS-1<br>*Pseudomonas aeruginosa* UCBPP-PA14<br>*Rhodopseudomonas palustris* TIE-1<br>*Rickettsia bellii* RML369-C<br>*Nostoc punctiforme* PCC 73102<br>*Petrotoga mobilis* SJ95<br>*Oligotropha carboxidovorans* OM5<br>*Pelobacter propionicus* DSM 2379<br>*Rickettsia rickettsii* str. Iowa<br>*Pseudomonas putida* F1<br>*Phenylobacterium zucineum* HLK1<br>*Pseudomonas fluorescens* Pf0-1<br>*Nitrosococcus oceani* ATCC 19707<br>*Pasteurella multocida* subsp. *multocida* str. Pm70<br>*Pseudomonas putida* W619<br>*Prochlorococcus marinus* subsp. *marinus* str. CCMP1375<br>*Mycoplasma penetrans* HF-2<br>*Roseobacter denitrificans* OCh 114<br>*Prochlorococcus marinus* str. AS9601<br>*Rickettsia peacockii* str. Rustic<br>*Nocardia farcinica* IFM 10152<br>*Neisseria gonorrhoeae* FA 1090<br>*Nostoc* sp. PCC 7120<br>*Photorhabdus asymbiotica*<br>*Rhodospirillum rubrum* ATCC 11170<br>*Rhodoferax ferrireducens* T118		

Rhizobium etli CIAT 652<br>*Prochlorococcus marinus* str. MIT 9313<br>*Pseudomonas entomophila* L48<br>*Nitrosomonas eutropha* C91<br>*Orientia tsutsugamushi* str. Ikeda<br>*Pelodictyon phaeoclathratiforme* BU-1<br>*Neorickettsia sennetsu* str. Miyayama<br>*Rhodococcus opacus* B4<br>*Pseudomonas syringae* pv. syringae B728a<br>*Pseudoalteromonas atlantica* T6c<br>*Psychrobacter cryohalolentis* K5<br>*Rhizobium* sp. NGR234<br>*Polynucleobacter necessarius* subsp. necessarius STIR1<br>*Pseudomonas syringae* pv. tomato str. DC3000<br>*Rhodopseudomonas palustris* BisB18<br>*Mycoplasma hyopneumoniae* J<br>*Pseudomonas putida* GB-1<br>*Rhodococcus erythropolis* PR4<br>*Renibacterium salmoninarum* ATCC 33209<br>*Pectobacterium carotovorum* subsp. carotovorum PC1<br>*Nitrosomonas europaea* ATCC 19718<br>*Rhodopseudomonas palustris* BisB5<br>*Nautilia profundicola* AmH<br>*Rhizobium leguminosarum* bv. trifolii WSM2304<br>*Mycoplasma pneumoniae* M129<br>*Psychromonas ingrahamii* 37<br>*Polynucleobacter necessarius* subsp. *asymbioticus*<br>QLW-P1DMWA-1		

Ralstonia solanacearum GMI1000<br>*Rickettsia massiliae* MTU5<br>*Polaromonas naphthalenivorans* CJ2<br>*Pelobacter carbinolicus* DSM 2380<br>*Pseudomonas aeruginosa* LESB58<br>*Neisseria meningitidis* MC58<br>*Rickettsia akari* str. Hartford<br>*Mycoplasma mobile* 163K<br>*Nitratiruptor* sp. SB155-2<br>*Pseudomonas aeruginosa* PA7<br>*Rhodopseudomonas palustris* HaA2<br>*Nitrobacter winogradskyi* Nb-255<br>*Polaromonas* sp. JS666<br>*Ruegeria pomeroyi* DSS-3<br>*Neisseria meningitidis* 053442<br>*Pectobacterium wasabiae* WPP163<br>*Neisseria meningitidis* Z2491<br>*Rhizobium leguminosarum* bv. *viciae* 3841<br>*Prochlorococcus marinus* str. NATL2A<br>*Rhodobacter sphaeroides* ATCC 17025<br>*Neisseria meningitidis alpha14*<br>*Shewanella baltica* OS155<br>*Salmonella enterica* subsp. *enterica serovar* Enteritidis str.<br>*Streptococcus pyogenes* MGAS9429<br>*Staphylococcus carnosus* subsp. *carnosus* TM300<br>*Sinorhizobium meliloti* 1021<br>*Streptococcus agalactiae* A909		

Staphylococcus aureus subsp. *aureus* JH9<br>*Streptococcus pyogenes* MGAS315<br>*Shewanella frigidimarina* NCIMB 400<br>*Stenotrophomonas maltophilia* K279a<br>*Shewanella putrefaciens* CN-32<br>*Staphylococcus aureus* subsp. *aureus* ED98<br>*Staphylococcus aureus* subsp. *aureus* Mu50<br>*Streptococcus pyogenes* M1 GAS<br>*Salmonella enterica* subsp. *enterica serovar* Choleraesuis str.<br>*Stenotrophomonas maltophilia* R551-3<br>*Streptococcus pyogenes* MGAS8232<br>*Staphylococcus aureus* subsp. *aureus* Mu3<br>*Saccharophagus degradans* 2-40<br>*Shewanella sp*. MR-4<br>*Shigella dysenteriae* Sd197<br>*Staphylococcus aureus* subsp. *aureus* MSSA476<br>*Shewanella sp*. MR-7<br>*Salmonella enterica* subsp. enterica serovar Agona str. SL483<br>*Shigella sonnei* Ss046<br>*Sinorhizobium medicae* WSM419<br>*Shewanella amazonensis* SB2B<br>*Sphingopyxis alaskensis* RB2256<br>*Streptococcus pneumoniae* R6<br>*Shewanella woodyi* ATCC 51908<br>*Staphylococcus aureus* subsp. *aureus* MW2<br>*Streptococcus equi* subsp. *equi* 4047		

Shewanella halifaxensis HAW-EB4<br>*Streptococcus pneumoniae* 70585<br>*Shewanella sediminis* HAW-EB3<br>*Salmonella enterica* subsp. *enterica serovar* Typhimurium str. LT2<br>*Shigella flexneri* 2a str. 301<br>*Streptococcus agalactiae* 2603V/R<br>*Streptococcus pyogenes* MGAS10270<br>*Streptococcus equi* subsp. *zooepidemicus* MGCS10565<br>*Staphylococcus aureus* subsp. *aureus* COL<br>*Staphylococcus aureus* subsp. *aureus* JH1<br>*Staphylococcus aureus* subsp. *aureus* MRSA252<br>*Streptococcus pyogenes* MGAS6180<br>*Staphylococcus aureus* subsp. *aureus* str. Newman<br>*Salmonella enterica* subsp. *enterica serovar* Paratyphi C strain<br>*Shewanella baltica* OS223<br>*Staphylococcus aureus* subsp. *aureus* NCTC 8325<br>*Streptococcus pneumoniae* CGSP14<br>*Shewanella oneidensis* MR-1<br>*Streptococcus pneumoniae* ATCC 700669<br>*Shigella boydii* CDC 3083-94<br>*Staphylococcus aureus* subsp. *aureus* N315<br>*Sodalis glossinidius* str. 'morsitans'<br>*Streptococcus pyogenes* MGAS10394<br>*Sphingomonas wittichii* RW1<br>*Salmonella enterica* subsp. *enterica serovar* Paratyphi A str. ATCC		

Streptococcus pyogenes NZ131<br>*Salmonella enterica* subsp. *enterica serovar* Newport str. SL254<br>*Shigella flexneri* 5 str. 8401<br>*Salmonella enterica* subsp. *enterica serovar* Typhi str. Ty2<br>*Salmonella enterica* subsp. *enterica serovar* Heidelberg str. SL476<br>*Salmonella enterica* subsp. *arizonae serovar* 62:z4,z23:--<br>*Staphylococcus saprophyticus* subsp. *saprophyticus* ATCC 15305<br>*Streptococcus mutans* UA159<br>*Staphylococcus epidermidis* RP62A<br>*Staphylococcus aureus* subsp. *aureus* USA300_TCH1516<br>*Streptococcus pneumoniae* Hungary19A-6<br>*Staphylococcus haemolyticus* JCSC1435<br>*Salmonella enterica* subsp. *enterica serovar* Schwarzengrund str.<br>*Streptococcus pneumoniae* TCH8431/19A<br>*Serratia proteamaculans* 568<br>*Staphylococcus aureus* RF122<br>*Streptococcus pneumoniae* D39<br>*Streptococcus* pneumoniae P1031<br>*Shewanella sp*. W3-18-1<br>*Streptococcus pneumoniae* TIGR4<br>*Streptococcus pneumoniae* Taiwan19F-14<br>*Streptococcus pneumoniae* G54<br>*Streptococcus pneumoniae* JJA		

Streptococcus pyogenes str. Manfredo<br>*Salmonella enterica* subsp. *enterica serovar* Paratyphi B str. SPB7<br>*Shigella flexneri* 2a str. 2457T<br>*Streptococcus pyogenes* MGAS2096<br>*Salmonella enterica* subsp. *enterica serovar* Paratyphi A str.<br>*Shewanella loihica* PV-4<br>*Streptococcus gordonii* str. Challis substr. CH1<br>*Salmonella enterica* subsp. *enterica serovar* Dublin str. CT_02021853<br>*Streptococcus equi* subsp. *zooepidemicus*<br>*Shewanella baltica* OS185<br>*Streptococcus pyogenes* MGAS5005<br>*Streptococcus pyogenes* SSI-1<br>*Shewanella pealeana* ATCC 700345<br>*Shewanella denitrificans* OS217<br>*Salmonella enterica* subsp. *enterica serovar* Gallinarum str. 287/91<br>*Staphylococcus epidermidis* ATCC 12228<br>*Streptococcus sanguinis* SK36<br>*Shewanella baltica* OS195<br>*Staphylococcus aureus* subsp. *aureus* USA300_FPR3757<br>*Shewanella piezotolerans* WP3<br>*Streptococcus pyogenes* MGAS10750<br>*Synechococcus sp*. JA-3-3Ab<br>*Thermoanaerobacter tengcongensis* MB4<br>*Xanthomonas oryzae* pv. *oryzae* PXO99A		

Yersinia pestis Antiqua<br>*Wigglesworthia glossinidia endosymbiont* of *Glossina brevipalpis*<br>*Ureaplasma parvum serovar* 3 str. ATCC 700970<br>*Thermotoga petrophila* RKU-1<br>*Streptomyces griseus* subsp. *griseus* NBRC 13350<br>*Wolbachia endosymbiont* of *Culex quinquefasciatus* Pel<br>*Yersinia pestis* KIM 10<br>*Zymomonas mobilis* subsp. *mobilis* NCIMB 11163<br>*Yersinia pseudotuberculosis* PB1/+<br>*Xylella fastidiosa* M12<br>*Thermosipho melanesiensis* BI429<br>*Thermosipho africanus* TCF52B<br>*Yersinia pestis* Angola<br>*Tropheryma whipplei* TW08/27<br>*Sulfurimonas denitrificans* DSM 1251<br>*Zymomonas mobilis* subsp. *mobilis* ZM4<br>*Xylella fastidiosa* 9a5c<br>*Streptomyces avermitilis* MA-4680<br>*Xanthomonas campestris* pv. *campestris* str. B100<br>*Xanthomonas oryzae* pv. *oryzae* KACC10331<br>*Xanthomonas campestris* pv. *vesicatoria* str. 85-10<br>*Thauera* sp. MZ1T<br>*Xylella fastidiosa* M23<br>*Verminephrobacter eiseniae* EF01-2<br>*Thiomicrospira crunogena* XCL-2<br>*Teredinibacter turnerae* T7901<br>*Yersinia pestis* Nepal516		

Streptococcus thermophilus LMD-9<br>*Wolbachia endosymbiont* strain TRS of *Brugia malayi*<br>*Streptococcus thermophilus* LMG 18311<br>*Tropheryma whipplei* str. Twist<br>*Xanthomonas axonopodis* pv. *citri* str. 306<br>*Xanthomonas campestris* pv. *campestris* str. 8004<br>*Sulfurovum* sp. NBC37-1<br>*Xanthomonas campestris* pv. *campestris* str. ATCC 33913<br>*Ureaplasma parvum serovar* 3 str. ATCC 27815<br>*Thermotoga maritima* MSB8<br>*Xanthomonas oryzae* pv. *oryzae* MAFF 311018<br>*Yersinia pestis* Pestoides F<br>*Ureaplasma urealyticum serovar* 10 str. ATCC 33699<br>*Tolumonas auensis* DSM 9187<br>*Synechococcus elongatus* PCC 6301<br>*Synechococcus* sp. RCC307<br>*Yersinia pestis* CO92<br>*Yersinia pseudotuberculosis* YPIII<br>*Thioalkalivibrio* sp. HL-EbGR7<br>*Yersinia enterocolitica* subsp. *enterocolitica* 8081<br>*Yersinia pseudotuberculosis* IP 31758<br>*Streptococcus thermophilus* CNRZ1066<br>*Thermotoga lettingae* TMO<br>*Thermotoga neapolitana* DSM 4359<br>*Synechococcus* sp. JA-2-3B'a(2-13)<br>*Trichodesmium erythraeum* IMS101<br>*Synechococcus* sp. CC9902<br>*Yersinia pseudotuberculosis* IP 32953		

Xylella fastidiosa Temecula1<br>*Thiobacillus denitrificans* ATCC 25259<br>*Yersinia pestis biovar* Microtus str. 91001<br>*Burkholderia xenovorans* LB400<br>*Vibrio vulnificus* YJ016<br>*Brucella melitensis* ATCC 23457<br>*Burkholderia ambifaria* AMMD<br>*Burkholderia pseudomallei* 1710b<br>*Pseudoalteromonas haloplanktis* TAC125<br>*Rhodobacter sphaeroides* ATCC 17029<br>*Vibrio cholerae* O1 *biovar* El Tor str. N16961<br>*Mycobacterium abscessus* ATCC 19977<br>*Cupriavidus taiwanensis*<br>*Vibrio fischeri* ES114<br>*Brucella canis* ATCC 23365<br>*Ralstonia pickettii* 12J<br>*Burkholderia pseudomallei* 1106a<br>*Cupriavidus metallidurans* CH34<br>*Vibrio vulnificus* CMCP6<br>*Shewanella* sp. ANA-3<br>*Brucella melitensis biovar* Abortus 2308<br>*Burkholderia cenocepacia* MC0-3<br>*Agrobacterium radiobacter* K84<br>*Burkholderia vietnamiensis* G4<br>*Burkholderia mallei* SAVP1<br>*Burkholderia thailandensis* E264<br>*Burkholderia pseudomallei* MSHR346<br>*Rhodobacter sphaeroides* 2.4.1		

Burkholderia mallei NCTC 10229<br>*Ochrobactrum anthropi* ATCC 49188<br>*Burkholderia phytofirmans* PsJN<br>*Leptospira borgpetersenii serovar* Hardjo-bovis JB197<br>*Vibrio splendidus* LGP32<br>*Brucella abortus* bv. 1 str. 9-941<br>*Agrobacterium vitis* S4<br>*Brucella microti* CCM 4915<br>*Paracoccus denitrificans* PD1222<br>*Leptospira borgpetersenii serovar* Hardjo-bovis L550<br>*Vibrio parahaemolyticus* RIMD 2210633<br>Burkholderia *pseudomallei* 668<br>*Leptospira* interrogans *serovar* Lai str. 56601<br>*Burkholderia cenocepacia* J2315<br>*Vibrio fischeri* MJ11<br>*Brucella suis* 1330<br>*Burkholderia phymatum* STM815<br>*Brucella melitensis* bv. 1 str. 16M<br>*Burkholderia multivorans* ATCC 17616<br>*Burkholderia glumae* BGR1<br>*Vibrio harveyi* ATCC BAA-1116<br>*Burkholderia* sp. 383<br>*Brucella ovis* ATCC 25840<br>*Burkholderia pseudomallei* K96243<br>*Arthrobacter* sp. FB24<br>*Streptococcus dysgalactiae* subsp. *equisimilis* GGS_124<br>*Vibrio cholerae* MJ-1236<br>*Leptospira biflexa serovar* Patoc strain 'Patoc 1 (Paris)'		

Burkholderia cenocepacia HI2424<br>*Ralstonia eutropha* H16<br>*Rhodobacter sphaeroides* KD131<br>*Burkholderia mallei* ATCC 23344<br>*Brucella abortus* S19<br>*Vibrio cholerae* O395<br>*Ralstonia pickettii* 12D<br>*Variovorax paradoxus* S110<br>*Brucella suis* ATCC 23445<br>*Ralstonia eutropha* JMP134<br>*Vibrio cholerae* M66-2<br>*Aliivibrio salmonicida* LFI1238<br>*Leptospira interrogans serovar* Copenhageni str. *Fiocruz* L1-130<br>*Burkholderia cenocepacia* AU 1054<br>*Leptospira biflexa serovar* Patoc strain 'Patoc 1 (Ames)'<br>*Burkholderia mallei* NCTC 10247<br>*Burkholderia ambifaria* MC40-6<br>*Cyanothece* sp. ATCC 51142<br>*Burkholderia pseudomallei* 1106a<br>*Vibrio harveyi* ATCC BAA-1116<br>*Burkholderia pseudomallei* 1710b<br>*Burkholderia mallei* SAVP1<br>*Burkholderia thailandensis* E264<br>*Burkholderia pseudomallei* 668<br>*Agrobacterium tumefaciens* str. C58<br>*Burkholderia mallei* NCTC 10247		
